# Distinct target cell-dependent forms of short-term plasticity of the central visceral afferent synapses of the rat

**DOI:** 10.1186/1471-2202-11-134

**Published:** 2010-10-20

**Authors:** Kiyofumi Yamamoto, Jun Noguchi, Chiaki Yamada, Ayako M Watabe, Fusao Kato

**Affiliations:** 1 Laboratory of Neurophysiology, Department of Neuroscience, Jikei University School of Medicine, Minato-ku, Tokyo 105-8461, Japan

## Abstract

**Background:**

The visceral afferents from various cervico-abdominal sensory receptors project to the dorsal vagal complex (DVC), which is composed of the nucleus of the solitary tract (NTS), the area postrema and the dorsal motor nucleus of the vagus nerve (DMX), via the vagus and glossopharyngeal nerves and then the solitary tract (TS) in the brainstem. While the excitatory transmission at the TS-NTS synapses shows strong frequency-dependent suppression in response to repeated stimulation of the afferents, the frequency dependence and short-term plasticity at the TS-DMX synapses, which also transmit monosynaptic information from the visceral afferents to the DVC neurons, remain largely unknown.

**Results:**

Recording of the EPSCs activated by paired or repeated TS stimulation in the brainstem slices of rats revealed that, unlike NTS neurons whose paired-pulse ratio (PPR) is consistently below 0.6, the distribution of the PPR of DMX neurons shows bimodal peaks that are composed of type I (PPR, 0.6-1.5; 53% of 120 neurons recorded) and type II (PPR, < 0.6; 47%) neurons. Some of the type I DMX neurons showed paired-pulse potentiation. The distinction of these two types depended on the presynaptic release probability and the projection target of the postsynaptic cells; the distinction was not dependent on the location or soma size of the cell, intensity or site of the stimulation, the latency, standard deviation of latency or the quantal size. Repeated stimulation at 20 Hz resulted in gradual and potent decreases in EPSC amplitude in the NTS and type II DMX neurons, whereas type I DMX neurons displayed only slight decreases, which indicates that the DMX neurons of this type could be continuously activated by repeated firing of primary afferent fibers at a high (~10 Hz) frequency.

**Conclusions:**

These two general types of short-term plasticity might contribute to the differential activation of distinct vago-vagal reflex circuits, depending on the firing frequency and type of visceral afferents.

## Background

The visceral afferents arising from various kinds of receptors carry a wide range of information about the status of the cervico-abdominal organs including gastric load [1], esophageal tension [2,3], lung volume [4-6], arterial blood pressure [7], chemosensory inputs from the carotid bodies [8] and intragastric concentrations of bioactive substances [1,9]. Compared to somatosensory sensation that reports rapid touch pressure changes and acute nociceptive information, these sets of visceral information are encoded in a long-lasting and slowly changing frequency-modulated series of action potentials and transmitted to the brain via primary afferent fibers that pass through the vagus and glossopharyngeal nerves. These afferent axons then form their first intracerebral synapses in the dorsal vagal complex (DVC) composed of the nucleus of the solitary tract (NTS), area postrema and the dorsal motor nucleus of the vagus nerve (DMX). The DVC is located on or close to the dorsal aspect of the medulla beneath the fourth ventricle [10-12]. In this regard, frequency-dependent transfer properties, such as short-term plasticity and frequency-dependent suppression [13,14], at these first synapses in the DVC should play the primary role in determining how the central neurons respond to frequency modulation-encoded visceral information [15].

Synaptic transmission between the baroreceptor afferents and the second-order neurons in the NTS has been well studied and shown to be strongly suppressed at elevated input frequencies in anesthetized rats [7] and in brainstem slice preparations [13,16-20]; i.e., neurons cannot respond to inputs at an elevated frequency or at short inter-spike intervals. One of the advantages of such "low-pass filter" characteristics of synaptic transmission is that it can attenuate excessive rapid fluctuations in central reflex responses of the autonomic output [7]. This is a property that is suitable for dealing with "phasic" inputs, whereas an apparent disadvantage is that neurons cannot faithfully respond to continued high-frequency "tonic" inputs. This is however apparently contradictory because a subset of vagal afferents shows continuous discharge at approximately 10 Hz in response to elevated gastric load [1] and esophageal tension [2,3].

Here, we analyzed the short-term plasticity of the synapses between the primary afferents and various types of DVC neurons in acute slice preparations. In particular, we compared the frequency dependence between the well-studied NTS neurons [7,13,16-20] and the much less studied DMX neurons [10,11,16,21-24] that also form a part of vago-vagal reflex pathway [25]. Our findings show that distinct classes of postsynaptic neurons show distinct types of short-term plasticity and frequency dependence resulting from distinct presynaptic mechanisms, depending on the type and function of each neuron.

## Results

### Distinct short-term plasticity at synapses between TS afferents and DVC neurons

**Figure 1:**
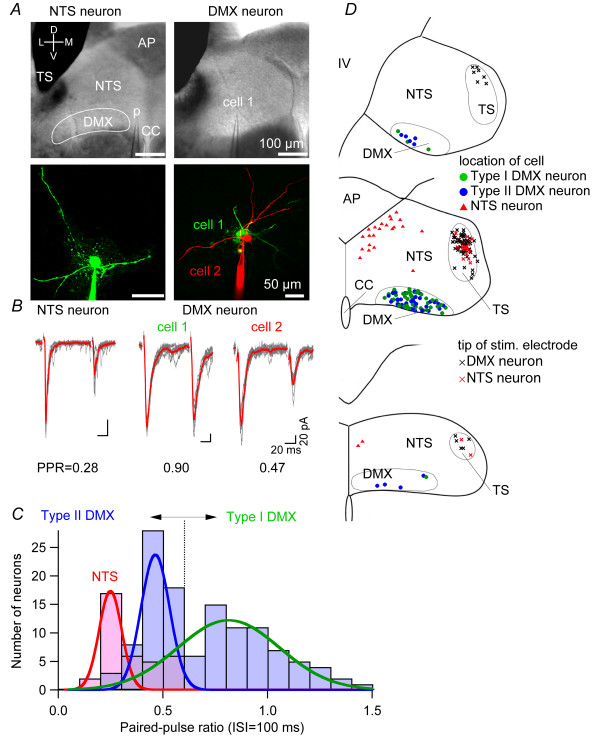
**Analysis of paired-pulse ratio of the excitatory transmission from the TS to DVC neurons**. *A*, above, IR-DIC photomicrographs indicating the location of the patch recording pipette (p) and the stimulation electrode in the DVC. Left, recording from a dm-cNTS (NTS) neuron; right, recording from a DMX neuron (designated as "cell 1"). Bottom, confocal microscopic images of a dm-cNTS neuron that is visualized with injected Alexa Fluor 488 (left) and two DMX neurons (cell 1, visualized with Alexa Fluor 488 and shown in green; cell 2, with Alexa Fluor 568 and shown in red). *B*, sample traces of the EPSC waveform evoked by TS stimulation. Gray traces show the eight overlaid traces of consecutive responses and the red traces show their average. Left, responses of the dm-cNTS neuron shown in (*A*); right, responses of adjacent DMX neurons shown in *A *(cell 1 and cell 2). The values on the bottom show the PPR calculated for these three neurons. *C*, a histogram showing the distribution of PPR of the DMX and dm-cNTS neurons. Bars in light red show the PPR for dm-cNTS neurons (*n *= 35) and those in light blue show the PPR for DMX neurons (*n *= 120). Note a trough of the histogram around PPR = 0.6, which is the threshold used to classify the DMX neurons into type I (PPR > 0.6) and type II (PPR < 0.6). All dm-cNTS neurons showed PPR < 0.6. Curves in red, blue and green show the best-fit Gaussian distributions representing the histogram peaks for dm-cNTS, type II DMX and type I DMX neurons, respectively. *D*, schematic drawings of the distribution and localization of the soma of recorded neurons (33 dm-cNTS, red triangles; 56 type I DMX, green circles; 52 type II DMX, blue circles) and the tip of stimulus electrodes (black crosses, for DMX neuron responses; red crosses, for dm-cNTS neuron responses), plotted on three representative coronal slices (from top to bottom, rostral to caudal).

The data reported below consist of results from recordings in 152 DMX and 42 neurons in the dorsomedial part of the caudal NTS (dm-cNTS) from 149 rats. First, we compared the short-term plasticity by analyzing the paired-pulse ratio (PPR) of EPSC amplitude between dm-cNTS neurons and DMX neurons. The PPR was calculated as the ratio of amplitudes of EPSCs evoked by two successive stimulations with an interval of 100 ms, indicating how amplitude of an EPSC is affected by an immediately preceding EPSC occurring at a short interval (i.e., an index of the short-term plasticity). Figure 1A shows typical examples of dm-cNTS neuron morphology (left) and two DMX neurons (right) visualized by intracellular Alexa Fluor injection (below), as well as corresponding IR-DIC images (above). Figure 1B presents EPSC traces recorded from the neurons shown in Figure 1A. In all dm-cNTS neurons analyzed, dm-cNTS neurons showed strong paired-pulse depression (e.g., PPR of the representative dm-cNTS neuron shown in Figure 1A and 1B was 0.28), in line with results reported previously [20,26]. The histogram of the PPR obtained from dm-cNTS neurons (red bars in Figure 1C) indicates that the PPR of dm-cNTS neurons is no greater than 0.6; the mean value was 0.35 ± 0.02 (n = 35) at an inter-stimulus interval of 100 ms, suggesting consistent paired-pulse depression.

In contrast, DMX neurons showed much larger variation in terms of short-term plasticity. For example, two representative DMX neurons, which were located immediately adjacent to each other in the same slice (cell 1 and cell 2 in Figure 1A-DMX neuron), showed distinct paired-pulse plasticity (Figure 1B); one with almost no paired-pulse depression (cell 1, Figure 1B-cell 1; PPR = 0.90) and another with strong paired-pulse depression (cell 2; PPR = 0.47). The histogram with blue bars in Figure 1C, constructed on the basis of recordings from 115 DMX neurons, further confirms that the distribution of PPR in DMX shows greater variation (ranging from 0.2 to 1.5) than that of dm-cNTS neurons. This distribution of PPR among DMX neurons was well fitted with a composite of two Gaussian distributions (blue and green lines in Figure 1C). We classified DMX neurons in two groups because: i) these two Gaussian distributions showed the smallest overlap at a PPR value around 0.6; ii) there was a trough at around 0.6 for the distribution of PPR among DMX neurons; iii) the PPR of dm-cNTS neurons never exceeded 0.6. The groups were as follows: those exhibiting PPRs larger than 0.6 ("type I" DMX neurons; 47.5% of 120 DMX neurons) and those with PPRs smaller than 0.6 ("type II" DMX neurons; 52.5%) (Figure 1C). According to this classification, cell 1 in Figure 1B was classified as a type I DMX neuron and cell 2 as a type II DMX neuron. The mean PPR values of type I and type II DMX neurons were 0.92 ± 0.02 (n = 63) and 0.46 ± 0.10 (n = 57), respectively. Visualization of cell forms with intracellular injection Alexa Fluor or Neurobiotin was made in 11 DMX cells, of which 5 type I and 6 type II neurons, and there was no apparent difference in the soma shape, the direction and the number of thick dendrites.

### Distinct short-term plasticity results from distinct properties of afferent inputs to each postsynaptic neuron

To our knowledge, this distinction with regard to short-term plasticity in DMX neurons has not been described before. These results were unexpected because it has been believed that DMX neurons, the majority of which are parasympathetic preganglionic neurons, represent a relatively homogeneous population [27]. The following four lines of evidence strongly support the notion that these differences are intrinsic to the properties of synaptic transmission between TS and each distinct neuron type and do not reflect experimental artifacts.

**Figure 2:**
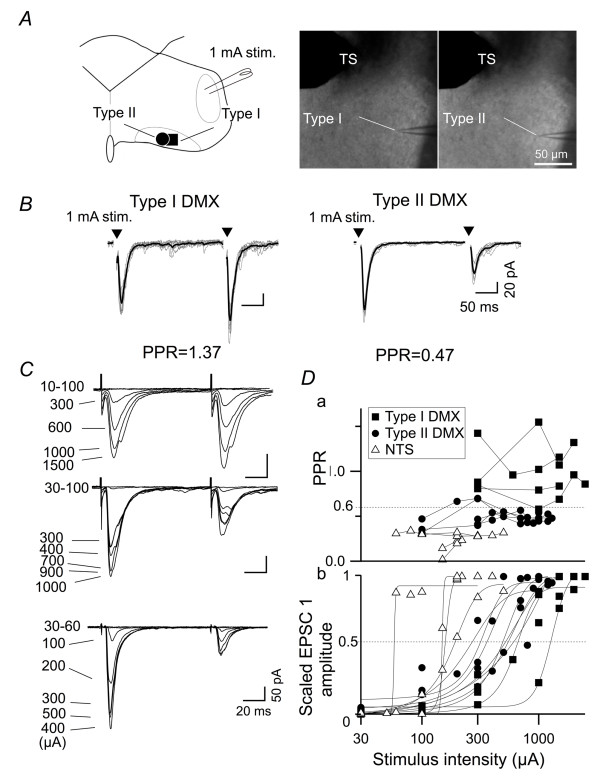
**Effect of stimulation protocol, electrode position and stimulus intensity on the PPR values**. *A*, left, schematic representation of the location of the two DMX neurons in which the EPSCs were evoked by stimulation at 1-mA intensity, which was delivered by the stimulating electrode placed on the TS. This electrode was never moved and the stimulation intensity was never changed during the recording of either DMX neuron. Right, IR-DIC images showing that two DMX neurons were located close to each other and the stimulation electrode on the TS was not moved. *B*, EPSC waveforms recorded from a type I (left) and type II (right) DMX neuron. Overlaid consecutive responses and the average trace (*n *= 8) in response to paired stimuli (arrowheads) at an interstimulus interval of 100 ms. Values on the bottom indicate the PPR. These results indicate that the distinct types of short-term plasticity in the DMX neurons do not depend on the intensity and site of stimulation. *C*, superimposed EPSC traces recorded from a type I DMX neuron (top), a type II DMX neuron (middle) and a dm-cNTS neuron (NTS; bottom) evoked by distinct stimulation intensities. EPSC_1 _and EPSC_2 _amplitudes gradually increased and reached the maximal amplitude. *D*, relationship between stimulus intensity and PPR (*a*) and normalized EPSC_1 _amplitude (*b*) that was measured in type I DMX (*n *= 4), type II DMX (*n *= 5) and dm-cNTS neurons (*n *= 4). Note that, despite marked increases in EPSC_1 _amplitude upon increasing stimulation intensity, the classification of neurons according to the PPR value was essentially unaffected, especially beyond the stimulus intensity that yielded 50%-EPSC_1 _amplitudes (50%-stimulus intensity).

First, this distinction does not result from differences in cell location, site of stimulation or condition of the slice because: i) type I and type II DMX neurons were intermingled at different levels of the rostrocaudal region in DMX (Figure 1D), and ii) both types of DMX neurons could be recorded within the same slice, without changing the intensity or site of stimulation (Figure 2A and 2B; an observation consistently repeated in three pairs of type I and II neurons recorded in three different slices from three different rats).

Second, this distinction between type I and type II DMX neurons was robustly observed at a wide range of stimulation intensities. To examine the hypothesis that a specific type of DMX neuron predominantly receives afferent fibers with a similar class of short-term plasticity (i.e., the type of postsynaptic neuron determines the type of afferents that converge), we analyzed the effect of changing the stimulation intensity from sub-threshold to supra-maximal levels on EPSC amplitude and PPR (Figure 2C) because this maneuver should gradually expand the population of activated afferents. As expected, augmentation of stimulation intensity increased the EPSC amplitude in all three groups, presumably by recruiting higher threshold primary afferent fibers. Compared to dm-cNTS neurons that showed abrupt increases in the EPSC amplitude upon increases in stimulation intensity [26], DMX neurons showed relatively gradual increases in amplitude (Figure 2D). The rate of rise of the intensity-amplitude curve estimated by sigmoidal curve fitting (Figure 2D) was significantly (Mann-Whitney U-test, *p *< 0.05) and markedly slower for DMX neurons than that for dm-cNTS neurons (dm-cNTS, type I and type II DMX neurons; 447 ± 282 mA^-1^, 5.2 ± 0.7 mA^-1 ^and 8.5 ± 1.5 mA^-1^, respectively); this suggests that the afferent fibers that converge onto the DMX neurons possess a larger variation in threshold than those that converge onto the NTS neurons. This limited convergence of afferents to the NTS neurons is consistent with the previous findings [26,28]. However, the intensity-PPR plot indicates that the PPR-dependent classification between NTS, type I and type II DMX neurons was relatively resistant to the changes in stimulation intensity (Figure 2D). This result argues against the possibility that the classification of type I and II DMX neurons is simply attributed to the properties of a set of fibers that are stimulated at a specific condition but supports the possibility that this classification is attributed to the type of the neuron of concern.

**Figure 3:**
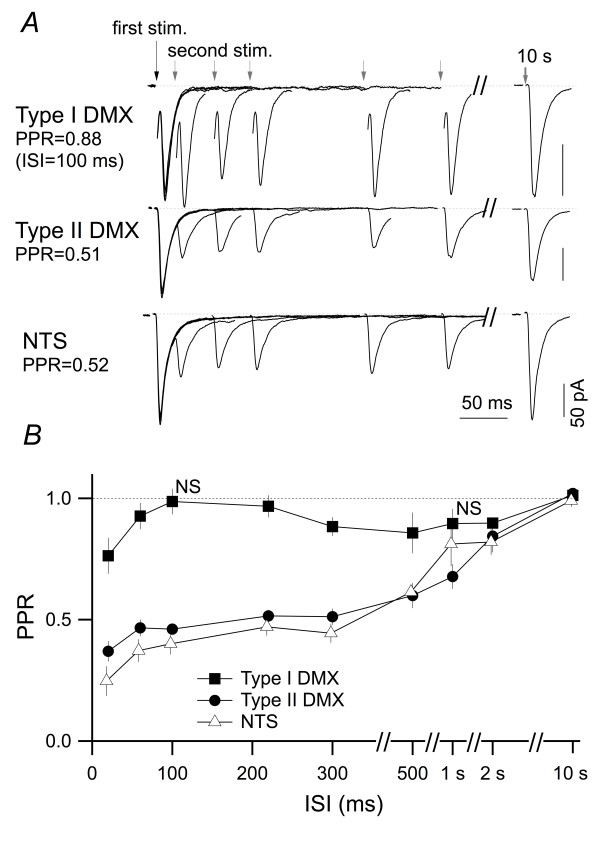
**Dependence of paired-pulse ratio of EPSC amplitude on interstimulus time intervals**. *A*, representative EPSC traces from type I DMX (top), type II DMX (middle) and dm-cNTS (NTS; bottom) neurons evoked by successive stimuli that were delivered at inter-stimulus intervals of 20, 60, 100, 220, 300 ms and 10 s. Seven traces are overlaid and aligned at the first EPSC waveform (long arrow at the left) for each neuron. Each trace is an average of 8 responses. *B*, summary of the paired-pulse ratio at different ISIs. Mean and SEM of the PPR values measured in type I (*n *= 4-19) and type II (*n *= 3-13) DMX neurons and dm-cNTS neurons (*n *= 4-9). Note that the ISI-dependence of PPR values also differed among these three neuron groups. The PPR values at each ISI were statistically compared to those at ISI of 10 s in each neuron group. At all time points, the PPR was significantly smaller (ANOVA; P < 0.05), except at 100 ms and 1 s for type I DMX neurons, at which the PPR was not significantly different from that at 10 s.

Third, the PPR-based distinction between different cell groups did not depend on the time between the two stimuli. The classification of type I and type II DMX neurons as shown in Figure 1C was determined according to the PPR with an inter-stimulus interval (ISI) of 100 ms at 2 mM [Ca^2+^]_o_. As has been commonly described [29,30], the PPR value changed as we changed ISI from 20 ms to 10 s (Figure 3). However, despite such ISI-dependent modulation of PPR, the ISI-PPR plot (Figure 3B) indicates that the PPR-dependent classification between NTS, type I and type II DMX neurons was relatively resistant to the changes in ISI. Indeed, the PPR for type I neurons was significantly larger than those for type II DMX neurons and dm-cNTS neurons at all ISI points from 20 ms to 500 ms (Kruskal-Wallis test; P < 0.05); this indicates that the marked difference in PPR between type I and II DMX neurons is a feature that is observed regardless of the interval between 20 ms and 500 ms. Interestingly, this strong paired-pulse depression observed in type II DMX and dm-cNTS neurons was associated with very slow recovery. Whereas the EPSC_2 _amplitude recovered to 95% of the EPSC_1 _at 70-ms ISI (estimated from the plot in Figure 3B) for type I DMX neurons, the complete recovery required 6.8 s and 8.1 s after EPSC_1 _for type II DMX and dm-cNTS neurons, respectively. Because the ≈90% recovery was attained at an ISI of 2 s (Figure 3B) for the dm-cNTS neurons, which is in a manner similar to the PPR-ISI curve reported in other studies for the dm-cNTS neurons [31], it is likely that this late recovery process after 2 s is distinct from the early recovery within the first 2 s. The mechanism underlying such slow recovery of the paired-pulse depression was not further analyzed in this study; however, maximal differences between type I and type II neurons in the PPR were observed at an ISI of 100 ms, which is a value used for classification in other parts of this study.

**Figure 4:**
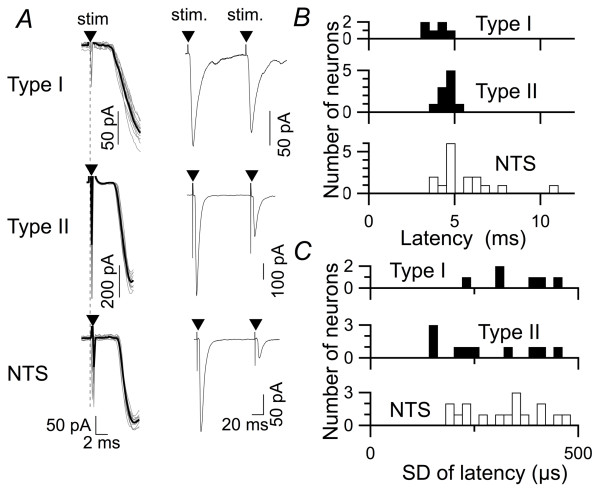
**EPSC latency in three neuron groups**. *A*, representative traces of EPSC_1 _evoked by TS stimulation (arrowheads). Left, averaged waveform (black; *n *= 11) and overlay of each waveform (gray) of the rising phase of EPSC_1 _responses in type I and type II DMX neurons and dm-cNTS (NTS) neurons; eleven consecutive responses. Note that the time between the stimulation and the beginning of downward deflections (latency) was constant across trials in these neurons. The latency values were 3.7, 4.0 and 4.3 ms for these type I DMX, type II DMX and dm-cNTS neurons, respectively. Right, averaged traces showing the responses to paired-pulse TS stimulation at an ISI of 100 ms. Note the different time scales between the left and right panels. The PPR values for these neurons were 0.86, 0.40 and 0.18 for type I DMX, type II DMX and dm-cNTS neurons, respectively.*B*, histogram showing the distribution of EPSC latency in type I (*n *= 6), type II DMX (*n *= 10) and dm-cNTS neurons (*n *= 16). The latency was measured in the EPSC recordings in which the onset of EPSC waveforms could be unequivocally defined, free of artifacts from the preceding stimulation.*C*, summary and comparison of the mean latency between type I and type II DMX and dm-cNTS neurons. The latency for EPSC in dm-cNTS neurons was slightly but significantly longer than that in DMX neurons. **, *p *< 0.01; one-way ANOVA.*D*, histogram showing the distribution of the SD of EPSC latency over 11 trials. *E*, summary and comparison of the standard deviation of latency among type I DMX, type II DMX and dm-cNTS neurons. There was no significant difference in the SD of latency for EPSC in these three classes of DVC neurons (one-way ANOVA).

Fourth, the latency, as well as the standard deviation of the latency, of type I and type II DMX neurons did not differ significantly. Their latency was consistently smaller than 6 ms (Figures 4A and 4B) without any significant difference between type I and II DMX neurons (*p *= 0.573; one-way ANOVA with Tukey post-hoc test), and the stimulation-to-onset time of EPSCs was highly consistent with little fluctuation (Figure 4A), as evidenced by consistently small SD for latency (consistently smaller than 500 μs in both type I and type II DMX neurons; Figure 4D). These data suggest that there are no apparently detectable differences in the processes from afferent activation to the activation of postsynaptic glutamate receptors between type I and II DMX neurons.

### Distinct short-term plasticity in DMX neurons is attributed to the variability in the probability of release from each fiber terminal

The above results clearly indicate that aspects of short-term plasticity vary among these three types of synapses of dm-cNTS and DMX neurons. We sought to identify distinct cellular mechanisms that underlie these differences in short-term plasticity.

**Figure 5:**
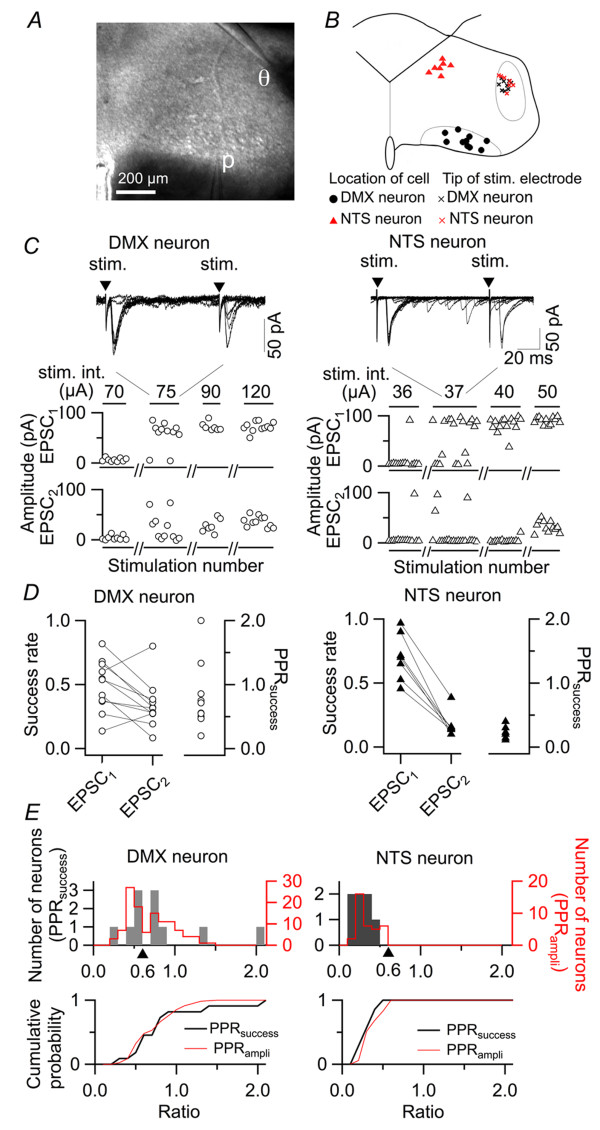
**Minimal stimulation demonstrated the monosynaptic nature of the TS-DMX and TS-dm-cNTS (NTS) transmission**. *A*, IR-DIC microphotograph showing the locations of a theta pipette bipolar electrode (θ) on the TS and recording pipette (p) in the DMX. *B*, summary of the location of the tip of the theta pipette bipolar electrode and recorded neurons. sfEPSC responses were recorded in the dm-cNTS (*n *= 7) and DMX (*n *= 11) neurons. The neurons shown here are not included in Fig. 1D. *C*, representative traces of sfEPSC in a DMX (left) and a dm-cNTS (right) neuron. Top, superimposed traces of EPSC waveforms that were evoked by minimal paired-pulse stimulation (arrowheads). The stimulation intensity was 75 μA for the DMX neuron and 37 μA for the dm-cNTS neuron. The plots on the bottom show the amplitude of sfEPSC_1 _and sfEPSC_2 _while increasing the stimulation intensity from one that evoked only failure responses to one that evoked mostly successful responses with only rare failures. *D*, success rate (defined as the ratio of non-failure events per trial) of EPSC_1 _and EPSC_2 _responses (the left-side ordinate) and PPR_success _(defined as the success rate of sfEPSC_2 _normalized by that of sfEPSC_1_; right-side ordinate) in the 8 DMX (left; open circles) and 7 dm-cNTS (right; filled triangles) neurons. *E*, histograms (top) showing the distribution of PPR_success _(filled gray bar; based on the sfEPSC recordings with minimal stimulation) and PPR_ampli _(the PPR estimated based on the amplitude ratio of EPSC_1 _and EPSC_2 _with submaximal stimulation; the same histograms shown in Fig. 1C) in DMX (left) and dm-cNTS (right) neurons, respectively. Cumulative probability curves (bottom) produced based on the histograms above. No significant difference was detected between the distributions of PPR_ampli _and PPR_success _in either DMX (*p *= 0.95) or dm-cNTS neurons (*p *= 0.40). Kolmogorov-Smirnov test.

To investigate if such distinct short-term plasticity results from specific characteristics of glutamate release from each primary afferent fiber, we measured the paired-pulse ratio of successful release from a single fiber by stimulating the TS with a theta-pipette-fabricated bipolar electrode with minimal stimulation intensity. This protocol elicits all-or-none single-fiber EPSC responses (sfEPSC) [32]. Figure 5 shows sfEPSC that were recorded in DMX and dm-cNTS neurons. In the DMX neuron shown in Figure 5C (left), the first stimulus successfully evoked sfEPSCs of almost the same amplitude and waveform, with only a few failures (2 of 11 trials) at the 75-μA stimulation intensity. The second stimulus more frequently failed to evoke sfEPSC (6 of 11 trials). Based on the failure rate, we defined "success rate" by subtracting the failure rate from unity. Then, based on the success rates that were estimated for the first and second sfEPSCs activated by two successive stimulations at 100-ms ISI, we estimated the paired-pulse ratio of the success rate (success rate 2 normalized by success rate 1), which is a single fiber version of the PPR of the EPSC amplitude, and designated it "PPR_success_" (when this is compared with the PPR of the EPSC amplitude, the latter is designated as "PPR_ampli_" below). In dm-cNTS neurons, the success rate for the first sfEPSC was much larger than that for the second sfEPSC, which resulted in a low PPR_success _(see Figure 5C right; PPR_success_, 0.28). The success rate varied between 0.2 and 0.8 for DMX neurons and 0.45 and 1.0 for dm-cNTS neurons (Figure 5D, Success rate). There was no significant difference between groups in the required intensity of minimal stimulation for the all-or-none responses (type I (PPR_success _> 0.6), 67.8 ± 13.3 μA (n = 6); type II (PPR_success _< 0.6), 69 ± 9.9 μA (n = 5); *p *= 0.96; *t*-test) and in the sfEPSC latency (type I, 7.6 ± 0.5 ms (n = 6); type II, 6.7 ± 0.8 ms (n = 5); *p *= 0.35; *t*-test).

The distribution of PPR_success _for the DMX was divided into two populations (< 0.6 and >0.6) in a similar manner to PPR_ampli_. There was no significant difference between the distributions of PPR_success _(gray bars) and PPR_ampli _(red cityscape plots) in the DMX and the dm-cNTS (Figure 5E; Kolmogorov-Smirnov test; *p *= 0.95 and *p *= 0.40 for the DMX and the dm-cNTS, respectively). There was no significant difference between the proportion of type I neurons, as classified according to PPR_ampli _and PPR_success _(according to PPR_ampli_, 63 neurons among 120 cells (52.5%); according to PPR_success_, 6 neurons among 11 cells (54.5%); *p *= 0.9, Chi-square test). These data also strongly support that the distinction between type I and II DMX neurons does not depend on the intensity of the stimulation. These results support the hypothesis that the paired-pulse plasticity observed in the amplitude of EPSC that is evoked by submaximal stimulation described above results mostly from short-term plasticity in the release probability and not from short-term plasticity in the postsynaptic AMPA receptor responses. These data indicate that the specific properties of short-term plasticity of the EPSC amplitude in different postsynaptic neurons result primarily from the properties of transmitter release from the presynaptic terminals.

### The difference in short-term plasticity between DMX neuron types is related to function

**Figure 6:**
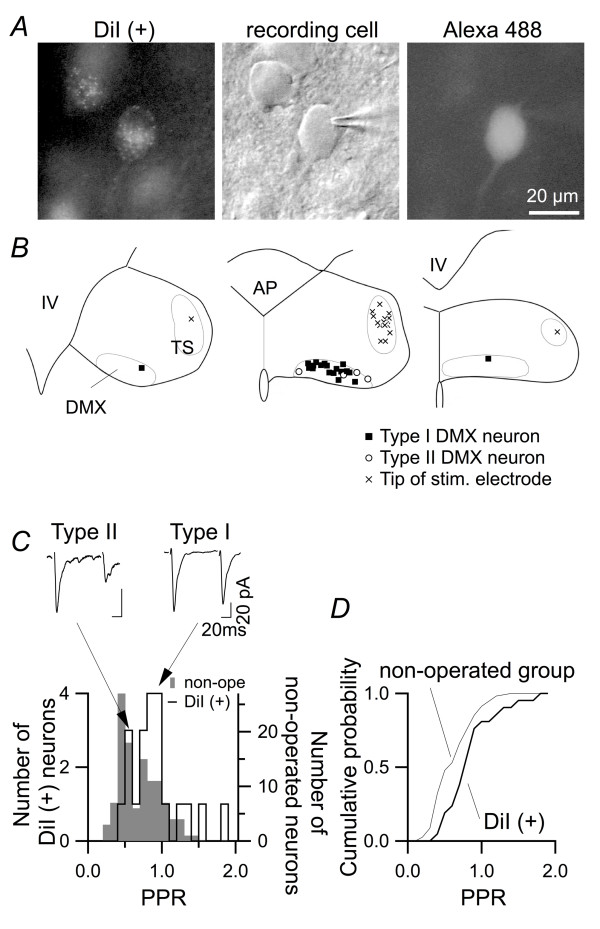
**Distribution of PPR of DMX neurons is related to the projection of the neuron**. *A*, identification of and targeted recording from a DMX neuron that projected to the anterior gastric branches of the subdiaphragmic vagus nerve, onto which DiI was placed. Left, epifluorescent image of the DiI fluorescence in the DMX; middle, IR-DIC image of the same observation field with a recording pipette on a DiI-positive neuron; right, fluorescence image of Alexa Fluor 488 injected into the identified neuron via internal solution. *B*, locations of the DiI-positive recorded DMX neurons (filled squares, DMX neurons classified as type I; open circles, those classified as type II) and the position of the tip of the stimulation electrode (crosses). *C*, distribution of PPR values among the DMX neurons. Gray bars: distribution of non-identified DMX neurons recorded from rats without DiI pretreatment (the same as in Fig. 1C); open cityscape histogram, that of DiI-positive DMX neurons. Insets above show averaged EPSC traces from two DiI-positive DMX neurons representing type I (left) and type II (right). *D*, cumulative probability plot based on the histogram in *C*. The distribution of PPR values of DiI-positive DMX neurons and that of DMX neurons recorded in the slices from non-operated rats were significantly different (Kolmogorov-Smirnov test, *p *= 0.035).

Next, we examined whether the two distinct types of short-term plasticity in DMX neurons are related to the functional roles performed by these neurons. A summary of the cell properties of dm-cNTS, type I and type II DMX neurons is shown in Table 1. When comparing type I and type II DMX neurons, most of the cell properties such as soma size, cell capacitance, and input resistance were not significantly different. Some of the characteristics, including the resting membrane potential, EPSC amplitude in response to submaximal stimulation and decay time-constant of EPSC, were only slightly but significantly different (Table 1). This result might imply that type I and type II DMX neurons belong to functionally distinct cell groups, a possibility that has not previously been proposed for the DMX population. It is generally challenging to address this issue, because unlike in the in vivo experiments, the function of each DMX neuron is difficult to examine directly in the isolated slice preparations and there is not yet any specific marker protein identified to distinguish distinct types of DMX neurons, if such subpopulations exist. Therefore, we addressed this question by examining whether the distribution of PPR values depends on the specific projection of the postsynaptic neuron recorded, on the assumption that the function of a DMX neuron should depend on the organ to which it projects. Based on this assumption, we placed DiI on the anterior gastric branches of the vagus nerve to identify the DMX neurons involved in gastric regulation and analyzed the short-term plasticity of these neurons (Figure 6A). The neurons showing DiI fluorescence at the soma (DiI(+) cells) were dispersed throughout the DMX (Figure 6B) and showed various PPR values from 0.42 to 1.80 (Figure 6C), indicating that both type I and type II DMX neurons belong to the DiI(+) population of cells. However, the DiI(+) cells exhibited a distribution of PPR values that differed significantly from that of the entire DMX neuron population (*p *= 0.035, Kolmogorov-Smirnov test); i.e., those projecting to stomach (n = 21) more frequently showed larger values of PPR and those with small PPRs (e.g., <0.5) were very rare compared to the PPR distribution based on whole DMX neurons recorded (n = 120; Figure 6D; c.f., Figure 1C). The proportions of type I neurons among the total population of DMX neurons (52.5% of 120 cells) and among those projecting to stomach (81.0% of 21 cells) were also significantly different (Fisher's exact probability test; *p *= 0.017). Table 2 summarizes the cellular and synaptic properties of DiI(+) DMX neurons, which showed significantly different distribution of input resistance and EPSC amplitude from that of whole DMX neurons recorded without preceding retrograde labeling (Kolmogrov-Smirnov test; *p *< 0.05), suggesting also a distinct excitability of DiI(+) DMX neurons. These results suggest that the distinction between type I and type II neurons is related to the projection target of each DMX neuron, presumably depending on its functional role. Another interpretation of these results is that abdominal operations and DiI placement on the vagus nerve resulted in selected reduction of fibers synapsing onto type II DMX neurons. This possibility cannot be fully ruled out with the present data; however, it also supports the notion that unidentified differences exist between types I and II DMX neurons with regard to afferent properties.

**Table 1:** Cell and synaptic characteristics of dm-cNTS and DMX neurons

	dm-cNTS neuron	DMX neuron		*p *value
		Type I	Type II	
Longitudinal soma size (μm)	13.0 ± 0.5 (31)	26.1 ± 0.5 (110)		<0.01^* 1^
		26.0 ± 0.6 (58)	26.2 ± 0.7 (52)	NS^2^
Resting membrane potential (mV)	-64.7 ± 2.2 (33)	-63.5 ± 0.9 (115)		NS^1^
		-65.5 ± 1.2 (59)	-61.3 ± 1.1 (56)	<0.05^** 2^
Cell capacitance (pF)	12.1 ± 1.1 (34)	31.2 ± 1.0 (117)		<0.01^* 1^
		31.0 ± 1.5 (61)	31.2 ± 1.5 (56)	NS^2^
Input resistance (MΩ)	755.6 ± 70.5 (34)	270.1 ± 11.6 (113)		<0.01^* 1^
		277.9 ± 17.1 (61)	261.0 ± 15.5 (52)	NS^2^
EPSC amplitude (pA) (submaximal stimulation)	243.5 ± 22.0 (35)	266.5 ± 14.1 (120)		<0.05^* 1^
		211.3 ± 14.5 (63)	323.2 ± 22.7 (57)	<0.01^**2^
Decay time-constant (ms)	6.0 ± 0.3 (35)	8.1 ± 0.2 (120)		<0.01^*1^
		8.6 ± 0.3 (63)	7.5 ± 0.3 (57)	<0.01^**2^
Time-to-peak (ms)	9.3 ± 0.3 (35)	9.4 ± 0.1 (120)		NS^1^
		9.6 ± 0.2 (63)	9.2 ± 0.2 (57)	NS^2^

Data are expressed as mean ± SEM. ^1^, comparisons were made between dm-cNTS and whole DMX neurons; ^2^, comparisons were made between type I and type II DMX neurons. NS, not significantly different. Numbers in parentheses indicate the number of neurons. Mann-Whitney U test.

**Table 2:** Cell and synaptic characteristics of DiI(+) type I and type II DMX neurons

	DiI(+) DMX neuron		*p *value
	Type I	Type II	
Longitudinal soma size (μm)	29.3 ± 1.3 (21)		NS^1^
	29.2 ± 1.4 (17)	30.0 ± 4.6 (4)	NS^2^
Resting membrane potential (mV)	-60.0 ± 1.1 (20)		NS^1^
	-60.6 ± 1.3 (16)	-57.75 ± 1.5 (4)	NS^2^
Cell capacitance (pF)	26.8 ± 1.8 (21)		NS^1^
	27.3 ± 2.2 (17)	24.9 ± 3.0 (4)	NS^2^
Input resistance (MΩ)	515.8 ± 59.0 (21)		<0.01*
	559.9 ± 63.4 (17)	328.0 ± 143.2 (4)	NS^2^
EPSC amplitude (pA) (submaximal stimulation)	114.0 ± 17.4 (21)		<0.01*
	110.4 ± 18.8 (17)	129.2 ± 62.4 (4)	NS^2^
Decay time-constant (ms)	8.5 ± 0.9 (21)		NS^1^
	7.7 ± 0.5 (17)	12.2 ± 1.0 (4)	NS^2^
Time-to-peak (ms)	9.9 ± 0.6 (21)		NS^1^
	9.3 ± 0.4 (17)	12.6 ± 0.2 (4)	NS^2^

Data are expressed as mean ± SEM. Numbers in parentheses indicate the number of neurons. NS^1^, the distribution of the values obtained from whole DiI(+) neurons was not significantly (*p *> 0.05) different from that from whole DMX neurons that were recorded without preceding retrograde labeling (Kolmogrov-Smirnov test). *, significantly different from the distribution of the values from whole DMX neurons. NS^2^, not significantly different between type I DMX and type II DMX neurons (*p *> 0.05).

### TS afferents form synapses with defined characteristics on distinct types of DMX neurons

**Figure 7:**
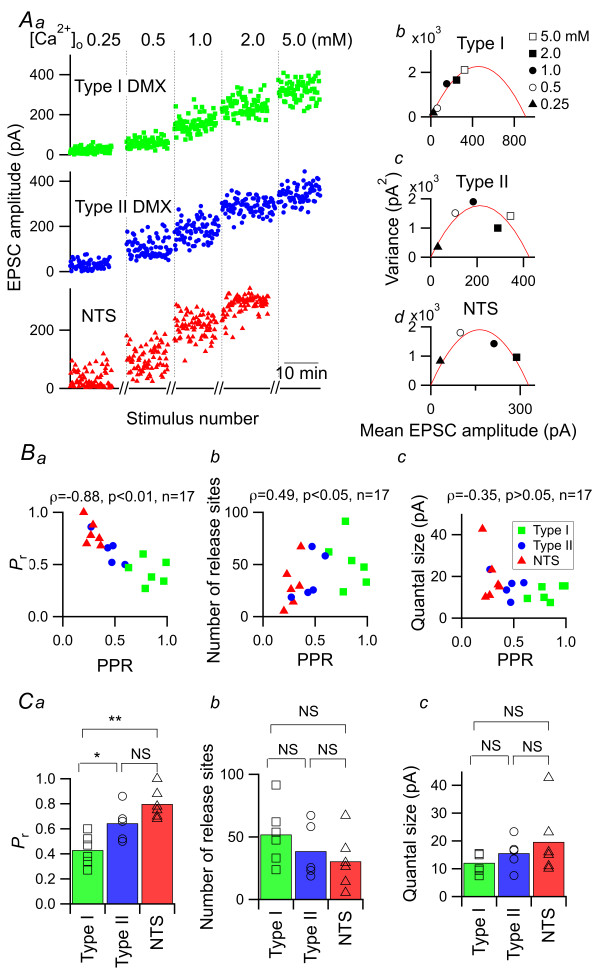
**Variance-mean analysis of the dm-cNTS (NTS) and DMX neurons revealed distinct synaptic properties**. *A*, variance-mean analysis of three representative neurons. Stable successive EPSC responses were recorded in the presence of 5.0 (shown for only DMX neurons), 2.0, 1.0, 0.5 and 0.25 mM [Ca^2+^]_o _solutions. *Aa*, the EPSC amplitude in response to each stimulation under different [Ca^2+^]_o _(shown above). Ab-c, the relationship between the mean EPSC amplitude (abscissa) and its variance (ordinates) over 70-94 consecutive trials. The quadratic functions of each graph show the best-fit estimation obtained by fitting the formula onto the actual values obtained in each neuron. *B*, the relationships between PPR and release probability (*P*_r_; *a*), number of release sites (*b*) and quantal EPSC size (*c*). Note the strong and significant correlation between PPR and *P*_r _(ρ = -0.88, *n *= 17, *p *< 0.01). *C*, summary of estimated *P*_r _(*a*), number of release sites (*b*) and quantal size (*c*). **, *p *< 0.01; *, *p *< 0.05; Mann-Whitney *U*-test. The same comparison was made for all other combinations in *Ca-c *but the values were not significantly different.

We sought to elucidate the mechanism that defines these differences between different pairs of pre- and postsynaptic neurons with regard to short-term synaptic plasticity. To address this issue, we performed mean-variance analysis of the EPSC amplitudes recorded at different release probabilities [26,33]. This method allows estimation of the number of release sites, quantal size and *P*_r _at a certain [Ca^2+^]_o _and has been successfully applied to central synapses [34], including those in the cNTS [26]. The mean EPSC amplitude and its variance were evaluated at different levels of [Ca^2+^]_o _ranging from 0.25-5 mM (Figure 7Aa). While the variance in amplitude was largest at 0.5 mM [Ca^2+^]_o _for this dm-cNTS neuron, the largest values were observed at 5 mM for the type I DMX neuron and 1 mM for the type II DMX neuron (Figures 7Aa-d). Theoretically, the relationship between amplitude and variance is best described by a quadratic function defined with *P*_r_, number of release sites, and quantal size [35]. Figure 7Ab-d shows the results of curve fitting for a representative neuron from each group shown in Figure 7Aa. The same estimation procedure was performed for a total of 17 neurons, in which EPSCs were recorded at different levels [Ca^2+^]_o _for a satisfactorily long period to allow for reliable estimations of variance. Figure 7Ba-c indicates the distribution of these variables in relation to the PPR of each neuron. As expected, the PPR value was negatively correlated with *P*_r _(at 2 mM [Ca^2+^]_o_; Figure 7Ba) and *P*_r _for type I DMX neurons was significantly smaller than those of type II DMX and dm-cNTS neurons (Figure 7Ca). Conversely, the number of release sites was positively correlated with the PPR (Figure 7Bb). There was no correlation between quantal size and PPR (Figure 7Bc). Estimated quantal size yielded similar values for different cell groups and was not correlated with the PPR of each neuron in any of these groups. These results imply that synapses with lower *P*_r _(i.e., larger PPR) are more closely associated with larger numbers of release sites, as is the case for type I DMX neurons.

### Distinct short-term plasticity defines the distinct frequency dependence of the DVC synapse

**Figure 8:**
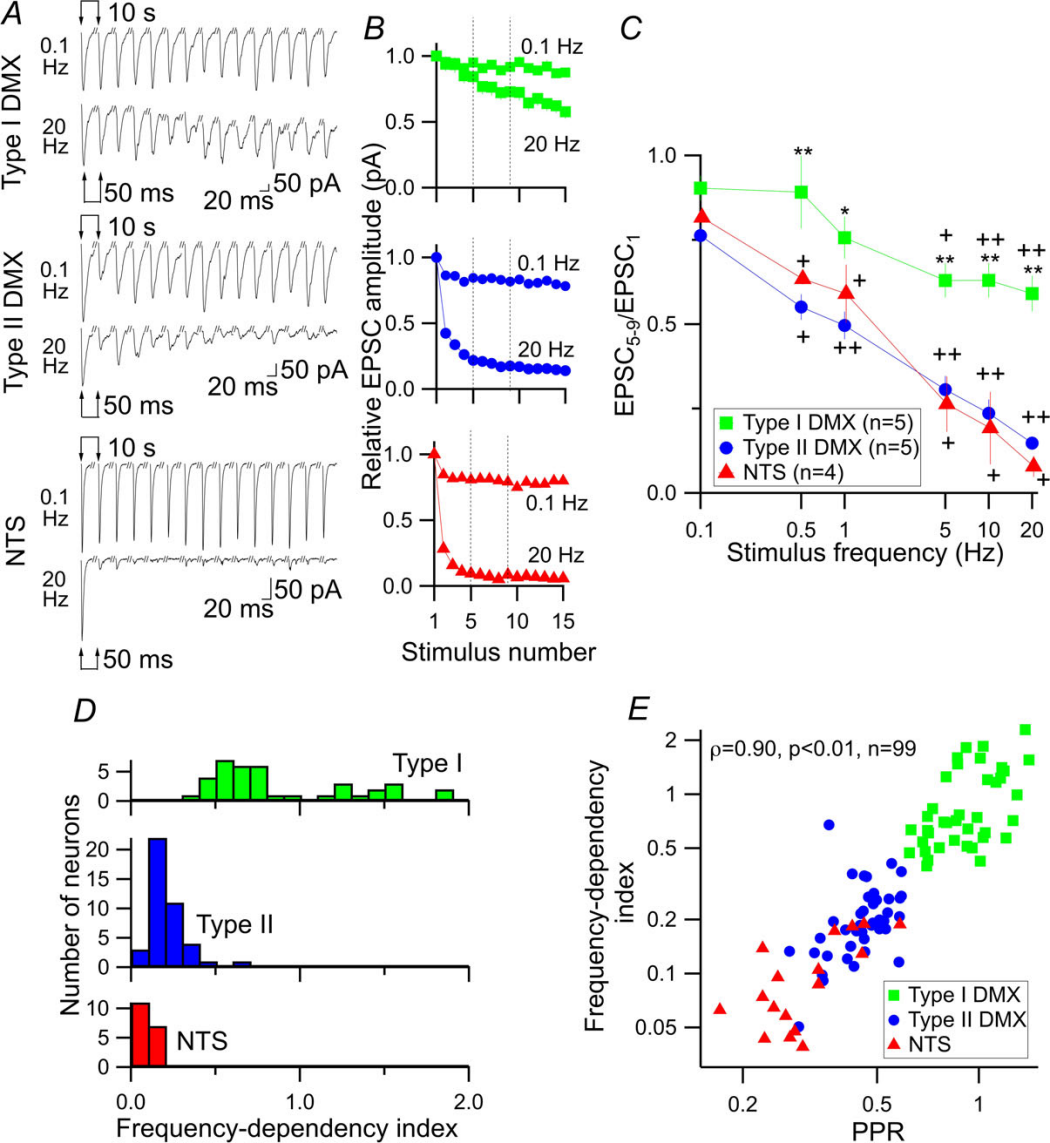
**Frequency dependence of EPSC following repetitive stimulation of the DVC neurons**. *A*, EPSC traces in response to repetitive stimulation at 0.1 Hz (top of each panel) and 20 Hz (bottom). Top to bottom: representative responses of type I and type II DMX neurons and a dm-cNTS (NTS) neuron. Traces in-between the responses were removed so that the changes in the EPSCs at different frequencies could be directly compared on the same time-scale. *B*, normalized EPSC amplitude at each stimulation (stimulus number 1-15) at 0.1 Hz and 20 Hz. Each marker and vertical bar represent the mean and SE of the values in 39 (type I DMX), 42 (type II DMX) and 18 (dm-cNTS) neurons. The length of the vertical SE bars was often smaller than the marker size, which made them masked behind the markers; this suggests fairly similar trajectories among neurons. Note the marked frequency-dependent depression at 20 Hz, especially in type II DMX and dm-cNTS neurons. Two vertical broken lines indicate the range of the 5th to 9th responses, in which typical depression patterns of the EPSC amplitude at 20-Hz stimulation were observed and used for evaluating the fdi. *C*, the depression of EPSC amplitude during the course of stimulation depended on the stimulation frequency. Abscissa, stimulus frequency; ordinate, the degree of EPSC amplitude depression as evaluated by the mean amplitude of EPSC_5 _to EPSC_9 _(5 EPSC responses), which was normalized to that of EPSC_1_. **, *p*< 0.01; *, *p *< 0.05; Kruskal-Wallis test to compare type I, type II DMX and dm-cNTS neurons; +, *p *< 0.05; ++, *p *< 0.01; Mann-Whitney test to compare the (EPSC_5-9_)/EPSC_1 _values at different stimulation frequencies with that at 0.1-Hz stimulation in each neuron group. *D*, distribution of fdi values estimated from recordings in type I DMX, type II DMX and dm-cNTS neurons. *E*, relationship between the PPR (abscissa) and fdi. Summary of data obtained for all cell types. Note the strong positive correlation between fdi and PPR (Spearman's ρ = 0.90, *p *< 0.01, *n *= 99).

The results above clearly indicate that, in the DVC, diverse forms of short-term plasticity characterize synapses between specific sets of pre-and postsynaptic neurons. These differing characteristics are determined by presynaptic release properties and depend on the type and projection of postsynaptic neurons. The vagal afferent fibers carry information arising from cervico-abdominal organs by modulating their firing frequency in a relatively low frequency range [1,2]. We asked whether such distinct and specific forms of short-term plasticity affect the frequency dependence of these synapses. To evaluate this frequency dependence, we repeatedly stimulated the primary afferents with 15 stimuli trains at 0.1 - 20 Hz (Figure 8A,B and 8C). As expected, type I DMX neurons showed limited depression during the course of repeated stimulation even when stimulated at 20 Hz (Figure 8B and 8C). In contrast, type II DMX and dm-cNTS neurons showed strong depression from the second stimulus onward, reaching a plateau value at the fifth stimulation (Figure 8B). In response to 20-Hz stimulation, we observed a slowly developing inward current in most of type I DMX neurons (88.6 ± 16.1 pA, n = 20) and to a smaller extent in type II DMX neurons (29.7 ± 4.1 pA, n = 25), which was presumably due to the accumulation of larger amplitude EPSC tails. Based on these observations, in order to quantitatively describe the frequency dependence of the depression during repeated stimulation, we calculated the mean EPSC amplitude over the fifth to ninth EPSCs (5 EPSCs) and this value was then normalized by the first EPSC amplitude (EPSC_5-9_/EPSC_1_; Figure 8C). The depression during repeated stimulation (as evaluated by EPSC_5-9_/EPSC_1_) was prominent in type II DMX and dm-cNTS neurons upon an increase in stimulation frequency (Figure 8C), whereas it was significantly higher at all frequency points from 0.5 to 20 Hz in type I DMX neurons (0.5-20 Hz, *p *< 0.05; Figure 8C). Then we defined the "frequency-dependency index" as follows: frequency-dependency index (fdi) = (EPSC_5-9_/EPSC_1 _at 20 Hz)/(EPSC_5-9_/EPSC_1 _at 0.1 Hz). This value indicates how stimulations at a high (20 Hz) frequency give rise to reductions in the EPSC amplitude during repeated stimulation compared to stimulation at a low (0.1 Hz) frequency. As shown in Figure 3, an ISI of 10 s (i.e., 0.1 Hz) was long enough for the amplitudes of successive EPSCs to recover. The mean fdi of type I and type II DMX neurons and dm-cNTS neurons were 0.91 ± 0.08 (n = 39), 0.21 ± 0.02 (n = 42) and 0.10 ± 0.01 (n = 18), respectively, and there was a significant difference in mean fdi and its distribution among three types of neuron groups (*p *< 0.01, Kruskal-Wallis test for mean; *p *< 0.0001, Kolmogorov-Smirnov test for the distribution). As expected, fdi values were highly correlated with the paired-pulse ratio of each neuron (Spearman's ρ = 0.90, *p *< 0.01, n = 99). These results indicate that the short-term plasticity analyzed above defines the properties of frequency-dependent synaptic transfer.

## Discussion

This study has described in detail, for the first time to our knowledge, distinct forms of short-term plasticity of the first intracerebral synapses formed between the visceral afferents and the second-order neurons in the DMX. Our data confirm neurophysiologically the previously published anatomical observations showing that the TS terminals make monosynaptic asymmetric contact with the dendrites of viscerally projecting DMX neurons in the cNTS regions [11,21,25]. Such a monosynaptic vago-vagal connection has also been functionally suggested by the authors of studies using slice preparations of rats and guinea pigs [16,22,24]. The novel finding of the present study is that, unlike the TS-NTS synapses that consistently exhibit strong short-term depression [13,26,36,37] and this study, the TS-DMX synapses show distinct types of short-term plasticity ranging from paired-pulse depression to facilitation, which results primarily from the distinct release properties of each afferent fiber.

### Diverse synaptic short-term plasticity of the TS-DMX synapses

In accordance with previously published studies [26,37], the TS-dm-cNTS transmission consistently exhibited a small PPR for EPSC amplitude, which is likely to result from paired-pulse depression of the release probability from single terminals, indicating the number of glutamate-containing vesicles recruited for exocytosis is reduced during repeated stimulation. In a similar manner to these dm-cNTS neurons [37-40], about half of DMX neurons were classified to "type II" DMX neurons that show short-term depression (PPR < 0.6) and another half were classified as "type I" DMX neurons exhibiting markedly higher PPR values (PPR > 0.6). To our knowledge, such short-term facilitation has never been reported with TS-DVC synapses.

The results of this study suggest that differences in short-term plasticity between type I and II DNX neurons result from the properties of vesicular release from the TS terminal, as has been demonstrated with TS-NTS synapses [26,41] as well as in this study. While the properties of the type II DMX synapses described in this study favor short-term depression primarily due to large *P*_r_, more frequent multi-vesicular release, and a smaller pool of docked vesicles [40,29,30], the properties of TS-type I DMX neuron synapses with much less short-term depression or rather facilitation in response to both submaximal and minimal stimulation are likely to result from small *P*_r _and a sufficient number of vesicles that are ready for exocytosis even after a short period of time. It will therefore be necessary for future studies to identify the molecular determinants of such distinct short-term plasticity in these DMX synapses [30].

### EPSCs in both types of DMX neurons are monosynaptically activated

To our knowledge, this study provides the first detailed description of the short-term plasticity of the TS-DMX synapses. Because the synaptic properties, especially those for TS-type I DMX transmissions, are distinctly different from those that have been demonstrated for TS-NTS synapses, it is essential to confirm that these transmissions are monosynaptic. For the monosynaptic transmission between arterial baroreceptor afferents and second-order cNTS neurons that receive these afferents at their perisomatic synapses, a pioneering study by Doyle & Andresen [13] has demonstrated that this transmission consistently shows smaller SD of latency over stimulation trials (the mathematical definition of a frequently used jargon, "jitter") in the horizontal slices at 34°C - 37°C. However, for the synapses between TS afferents and DMX neurons are formed on distant dendrites, and not on the soma [11,21]. Because, to our knowledge, there is no information on the SD of latency for synaptic transmission of the non-baroreceptor TS afferents that form synapses on the dendrites of the second-order DVC neurons, we could not use the smaller SD of latency as an *a priori *criterion for monosynaptic transmission. Whatever the case, the SD of latency in this study for the DMX and dm-cNTS neurons was consistently smaller than 500 μs (Figure 4D), which is a value used by Chen et al. [20] for the criteria for monosynaptic transmission in their study using a similar type of coronal slices and room temperature recording.

Instead, the following two pieces of evidence support the notion that the EPSCs in both type I and II DMX neurons are monosynaptically generated and not caused subsequently by the firing of second-order interneurons. First, there was no significant difference between groups in the latency of sfEPSC and EPSC, which argues against the possibility that one cell type involves the activation of more synapses than the other. If this was the case, it would be expected that polysynaptically evoked EPSCs would exhibit longer latency due to the time required for EPSP accumulation and the generation and conduction of action potentials in second-order interneurons. Such a pattern was not observed. Second, there was no significant difference between the required intensity of minimal stimulation for the all-or-none responses (i.e., sfEPSC); a result that argues against the possibility that minimal stimulation directly elicited interneuron firing by directly stimulating the somatodendritic regions of such interneurons in the vicinity of the stimulation electrode. If this possibility is true for any of these neuron groups, higher stimulation intensity should have been required to generate action potentials, due to their lower expression density of voltage-dependent Na^+ ^channels and lower specific membrane input resistance than in the axons. In addition, if firings of second-order NTS neurons underlie the EPSCs in the DMX cells, then the required minimal stimulation intensity should be higher so that the EPSP in the second-order neurons becomes large enough to generate action potentials by spatial summation in response to single-fiber stimulation. On the basis of these arguments, we conclude that both responses in type I and II DMX neurons occur through monosynaptic activation of neurons that are postsynaptic to the TS afferents.

### Short-term plasticity provides the basis for target-dependent frequency filters and optimal autonomic regulation

In a physiological sense, two pulses separated by a few hundred ms, as used in the PPR measurements, do not have any functional meaning. Rather, such short-term plasticity plays an essential role in determining the cut-off frequency of a synapse, thus limiting the content of information flow at a specific pathway [32,30,42-45]. The most important consequences of the higher PPR of type I DMX neurons are that these neurons are capable of being activated by repeated high-frequency afferent inputs up to a few tens of Hz in a manner that contrasts with that of the dm-cNTS and type II DMX neurons that filter out high-frequency components and can be sufficiently excited only by low (~0.1 Hz) frequency inputs. In contrast to paired pulse stimulation, continuously repeated action potentials might trigger other mechanisms that additionally affect synaptic depression, such as the activation of presynaptic metabotropic glutamate receptors [14] or adenosine receptors [46]. These possibilities are not explored in detail in the present study and are the subjects of future studies.

As a consequence, the findings of this study could be used to elaborate a "synaptic filter" model of these TS-DMX synapses. 1) At TS-type I DMX neuron synapses, the cut-off frequency [14] is elevated through a decreased release probability. 2) At TS-type II DMX neuron and TS-dm-cNTS neuron synapses, the cut-off frequency is lowered through increased release probability and short-term depression.

One of the possible functional implications of different forms of short-term plasticity would be the ability to filter the information represented by a series of repeated firings from the afferents arising from visceral organs depending on the discharge frequency. Such a mechanism could facilitate the frequency-dependent decoding of the afferent information to optimize the direct vago-vagal loop regulation of the visceral organs. Type I DMX neurons might receive afferents from so-called "dynamic range" receptors that start to generate a tonic series of action potentials at a low level of distention of digestive walls or at a low concentration of intragastric substances, such as cholecystokinin [1]. In contrast, type II DMX neurons might receive afferents arising from low-frequency-discharge receptors and/or selectively transmit information related to the "onset" of changes in the visceral environment. In these senses, type I DMX neurons can be denoted "tonic" receivers and type II neurons "phasic" receivers. Such distinct frequency filter properties of the first-step synapses of visceral afferents might contribute to the formation of appropriate discharge patterns of the neurons in the central circuit in response to various forms of afferent inputs, including those activated by duodenal acidification, intestinal distension, antral distension, cytokine generation during sepsis, and poisoning with emetics [47].

## Conclusions

To our knowledge, the frequency dependence and short-term plasticity of the TS-DMX synapses have not previously been reported; therefore, it has been considered, based on the data on the TS-dm-cNTS synapses, that the synaptic transmission between the TS and DVC neurons does not transfer high-frequency components and is therefore high-cut filtered. Nevertheless, this is not always the case because our first description of the frequency dependence of TS-DMX transmission presented here clearly indicates that there are distinct forms of short-term plasticity and filtering properties in these synapses. These results provide another example of pre- and postsynaptic functional association in which the role and type of a postsynaptic neuron determines the mode and characteristics of transmitter release from the presynaptic terminal that forms synapses onto it.

## Methods

### Slice preparation

Animal handling conformed to the Guiding Principles for the Care and Use of Animals in the Field of Physiological Sciences of the Physiological Society of Japan (1988) and was approved by the Animal Care Committee of The Jikei University. Transverse brainstem slices from Wistar rats (2-5 weeks) were prepared according to a method described previously [48,49]. Briefly, following the decapitation under sufficient anesthesia with overdose ketamine (100-150 mg kg^-1^, i.p.; in 133 rats) or isoflurane (5% in 100% O_2_; in 91 rats), the lower brainstem was dissected out and secured on the cutting stage of a vibrating blade slicer (DSK-1000, Dosaka EM, Kyoto, Japan) with the caudal end upward. As there was no apparent difference between the use of ketamine compared to isoflurane for the initial anesthesia, the data from both groups are pooled. Two to three transverse slices (400-μm thick) containing the DVC were cut in ice-cold "cutting" artificial cerebrospinal fluid (ACSF) composed of (in mM) NaCl 125, KCl 3, CaCl_2 _0.1, MgCl_2 _5, NaH_2_PO_4 _1.25, D-glucose 10, L-ascorbic acid 0.4 and NaHCO_3 _25 (pH 7.4 bubbled with 95% O_2 _+ 5% CO_2_; osmolarity, approximately 310 mOsm kg^-1^). The use of coronal transverse slices was necessary because of the following: 1) the DMX neurons could be clearly distinguished by microscopic observation from those in the adjacent NTS and the hypoglossal nucleus [50]; 2) TS fibers (which are dorsally located in the DVC) and DMX neuron soma (which are ventrally located) are well conserved in a single slice; and 3) recordings from the dm-cNTS and the DMX neurons without repositioning of the stimulation electrode in the same slice are possible, which allows for direct comparison of the synaptic characters between them. All of these are impossible or extremely difficult in horizontal slices [26]. We used the caudal part of the NTS (cNTS) that is mostly involved in circulatory, respiratory and digestive functions [51]. The slices were first incubated in a holding chamber with "standard" ACSF (the concentrations of CaCl_2 _and MgCl_2 _in the cutting ACSF were changed to 2 mM and 1.3 mM, respectively) at 37°C for 30-45 min and then kept at room temperature (25°C) in the same chamber for 0.5-8 hr until recording. One slice was transferred to a recording chamber (approximately 0.4 ml volume), submerged and continuously superfused with standard ACSF at a rate of 1-2 ml/min.

### Postsynaptic current recording

The whole-cell transmembrane current was recorded from small second-order dm-cNTS neurons and DMX neurons that were visually identified with an upright microscope (BX-50, Olympus) with infrared differential interference contrast optics (IR-DIC). The reason for the selective recording from the dorsomedial part of the cNTS was threefold. First, this part contains (but not exclusively) a large number of neurons receiving afferents from gastrointestinal regions [11], which might be suitable to compare with DMX that also receives mostly (but not exclusively) the gastrointestinal afferents. Second, this part roughly corresponds to "area 1" defined by Okada et al. [52] in which small neurons expressing VGLUT2 and GAD67 are localized, allowing comparison with a limited and defined group of cNTS neurons. Third, this part is similarly distant from the TS, at which the stimulation electrode was positioned, to that for the DMX, allowing the direct influence of stimulation current flow to the neuron being recorded to be similar and minimum. Neurons showing DiI fluorescence were identified prior to electrophysiological recordings using fluorescence microscopy [53]. Patch-clamp electrodes were made of borosilicate glass pipettes (1B120F-4, World Precision Instruments). The composition of the internal solution was (in mM) 120 potassium gluconate, 6 NaCl, 1 CaCl_2_, 2 MgCl_2_, 2 ATP magnesium, 0.5 GTP sodium, 5 EGTA, 10 HEPES hemisodium, 12 phosphocreatine disodium (pH was adjusted to 7.2 with KOH; osmolarity, ~303 mOsm kg^-1^). In one subset of experiments, Alexa Fluor 488 or 568 (Invitrogen) was added to the internal solution and fluorescence was visualized with a confocal scanning microscope (FV300, Olympus, Tokyo, Japan). In another series, neurobiotin (5 μg/μl; Vector Laboratories) added to the internal solution and the fluorescence was visualized following reaction with streptavidin-conjugated AlexaFluor 488 (Invitrogen) after fixation with 4% paraformaldehyde in 0.02 M PBS overnight at 4°C. The tip resistance of the electrode was 4-8 MΩ with the intracellular solution above. Neurons with either break-in membrane potential above -50 mV or non-overshooting action potential were discarded, but such neurons were quite rare in our slice preparations and IR-DIC-based pre-selection of healthy-looking cells. The longitudinal soma size (Table 1) was measured on the basis of the IR-DIC images focused at the circumference of the soma (e.g., Figure 6A, middle), captured at 0.336-μm/pixel resolution (640 pixel by 480 pixel), and stored on a computer, therefore providing a rough estimate of the soma diameter projected on the coronal plane. All DMX neurons showed smaller-or-larger delayed excitation upon depolarization from a hyperpolarized level [22]. The membrane current was recorded with an Axopatch 200B amplifier (Axon Instruments), low-pass filtered at 2 kHz and sampled at 4-20 kHz with a PowerLab interface (ADInstruments) together with the holding potential, stimulation timing pulse and TTL pulse from the electromagnetic valve controller used for drug application (VC-6, Warner Instruments). The membrane potential was held at -70 mV (liquid junction potential compensated) during the voltage-clamp recordings. All recordings were made at room temperature (20-25°C).

### TS stimulation

The tip of a bipolar concentric electrode (interpolar distance, 100 μm) was placed on the TS (Figure 1A). The distances between the soma of the recorded neurons and the tip of the stimulation electrode were 546.9 ± 22.1 μm and 414.1 ± 7.7 μm for dm-cNTS and DMX neurons, respectively (mean ± SEM; dm-cNTS, n = 33, range, 216.9-571.6 μm; DMX, n = 105, range 277.7-596.7 μm; as measured based on the digitally captured images of the slice with a spatial resolution of 0.336 μm/pixel). The short-term plasticity of all neurons recorded was evaluated using the paired-pulse ratio (PPR), the ratio of the EPSC amplitude evoked by the second stimulus (designated as EPSC_2_) to that evoked by the first stimulus (EPSC_1_), in response to two stimuli delivered to the TS with a 100-ms inter-stimulus interval (ISI). This paired-pulse stimulation was delivered at an interval of 10 s according to the recovery time course of the paired-pulse depression (See Figure 3). In a series of experiments, ISI was varied from 20 ms to 10 s (e.g., Figure 3). The intensity of the stimulation was set at the minimum that yielded a submaximal EPSC amplitude (0.01-2.5 mA, 100 μsec; called as "submaximal stimulation"; see Figure 2D). Because this pulse width of 100 μs is smaller than or equal to the sampling interval of the data acquisition, the stimulus artifacts are not faithfully represented by the digitally converted traces and therefore the artifacts in the traces are digitally masked in the figures. To evaluate the "frequency-dependency index" (fdi) of the synapse between the TS and the recorded neuron, the TS was stimulated at 0.1, 0.5, 1, 5, 10 and 20 Hz for 15 pulses at 1-mA intensity. The same stimulation protocol was carried out 3-4 times. The fdi was defined as follows: first, the mean amplitudes of five consecutive responses (EPSC_5 _to EPSC_9_) were normalized to EPSC_1 _in response to TS stimuli repeated at 20 Hz and 0.1 Hz. Then, their ratio (mean relative EPSC_5-9 _at 20 Hz divided by that at 0.1 Hz) was defined as fdi. This value represents the reduction in the EPSC amplitude after the 5th stimulus when stimulated at 20 Hz compared to stimulation at 0.1 Hz. In order to record postsynaptic responses evoked by an action potential generated in a single fiber located precisely at the place of stimulation, we performed "minimal" bipolar stimulation [32]. The stimulation pipettes were fabricated from theta capillaries (WPI) with similar pulling procedures to those used to fabricate patch electrodes (tip diameter, ~1-2 μm), which were filled with ACSF and placed on the TS (see e.g., Figure 5). The stimulus intensity was gradually increased to a fixed value at which EPSC appeared in an all-or-none manner with consistent amplitude and waveform (usually 20 -120 μA).

### Retrograde tracing of the gastric projection

Twenty-two Wistar rats (10-15 days-old) were lightly anaesthetized in an anesthesia box filled with with diethyl ether vapor for initial sedation for a few tens seconds and mounted in a prone position. Rats underwent subdiaphragm laparotomy under continuous isoflurane (1.5-2% in 100% O_2_) inhalation through a facial mask. Rats breathed spontaneously. Only when a spontaneous movement was detected, the concentration of isoflurane was eventually increased to 3%. The anterior gastric branches of the vagus nerve were isolated and a part (1-2 mm-length) of the nerve was covered with a cylinder-shaped silicone tube (inner diameter of 0.4 mm), inside of which was filled with1,1'-dioctadecyl-3,3,3',3'-tetramethylindocarbocyanine perchlorate (DiI) crystals mixed with DiI gel (Invitrogen, NeuroTrace DiI tissue-labeling paste), and the both ends of this tube were closed also with dental silicone (GC Corporation, Tokyo, Japan; Exafine injection) and the wound was sutured with 5-0 silk threads. The DVC slices were made 10-15 days after the operation. The DiI-positive cells were identified with epifluorescence optics for targeted patch-clamp recordings (e.g., Figure 6).

### Estimation of release probability (P_r_), total number of release sites (N) and quantal EPSC (q)

The variance (V) and the mean (M) of the amplitude of TS stimulation-evoked EPSCs over 49-96 trials (stimulation at every 10 s) recorded at different [Ca^2+^]_o _(0.25, 0.5, 1.0, 2.0 and 5.0 mM) were calculated. Assuming that the number of vesicles released from N release sites is governed by a binominal distribution, V is expressed as a quadratic function of M as follows: V = qM-M^2^/N, where q is the quantal postsynaptic response evoked by a single vesicular release. With theoretical constraints that V = 0 at M = 0 (zero variance and zero mean amplitude when no vesicle is released) and M = Nq (each whole N release site releases one vesicle, each causing q), the N and q are estimated by fitting this function on actual values obtained at different release probabilities (i.e., at different [Ca^2+^]_o _[26]. The sum of [Ca^2+^]_o _and [Mg^2+^]_o _was kept constant when manipulating [Ca^2+^]_o_. There is no existing mathematical method to examine the applicability of this method to actual synapses in the central nervous system that contain multiple release sites with heterogeneous properties. However, we consider that the synaptic transmission is well represented by this model because, in any of the neurons examined, the values obtained from experiments were well fitted by the theoretical quadratic function above with little deviation (see Figure 6Ab-d).

### Drugs

Picrotoxin (100 μM), strychnine HCl (1 μM) and MK-801 (20 μM) were added to the ACSF in all experiments to block GABA_A/C_, glycine and NMDA receptors, respectively. In a subset of the experiments, tetrodotoxin citrate (TTX, 1 μM) was also added to the ACSF after the recording of evoked postsynaptic currents to calculate the artifact waveform, which was subtracted from the averaged postsynaptic responses recorded in the absence of TTX. TTX, picrotoxin, strychnine hydrochloride, adenosine, and (+)MK-801 were purchased from Sigma. Other compounds were purchased from Sigma and Wako Pure Chemical Industries (Osaka, Japan).

### Data analysis and statistics

The recorded membrane current was analyzed off-line with the procedures written by F.K. running on Igor Pro 5 (WaveMetrics, Oregon, USA). The values are expressed as mean values ± standard error of the mean (S. E. M.). Differences in the values were compared with the Mann-Whitney *U*-test, the Kruskal-Wallis test, the ANOVA test, t-test or the Kolmogorov-Smirnov test (for distributions comparison). Differences with a probability (*p*) less than 0.05 were considered significant.

## Abbreviations

ACSF: artificial cerebrospinal fluid; AP: area postrema; CC: central canal; cNTS: caudal part of the nucleus of the solitary tract; DiI: 1,1'-dioctadecyl-3,3,3',3'-tetramethylindocarbocyanine perchlorate; dm-cNTS: dorsomedial part of the caudal nucleus of the solitary tract; DMX: dorsal motor nucleus of the vagus nerve; DVC: dorsal vagal complex; fdi: frequency-dependency index; IR-DIC: infrared differential interference contrast; ISI: inter-stimulus interval; IV: the fourth ventricle; *M*: mean; *N*: number of release sites; PPR: paired-pulse ratio; PPR_SUCCESS_: paired-pulse ratio of the success rate; PPR_ampli_: paired-pulse ratio of the EPSC amplitude; *P*_r_: release probability; q: quantal EPSC; sfEPSC: single-fiber EPSC; TS: the solitary tract; *V*: variance; XII: hypoglossal motor nucleus.

## Authors' contributions

Conception and design of experiments: KY and FK. Collection of data: in vitro experiments, KY; in vivo experiments, CY and KY. Analysis and interpretation of the data: KY, JN, AMW and FK. Initial draft: KY; Editing, revising, critical reading and completion of the manuscript: KY, JN, AMW and FK. The final version was read and approved by all authors.
